# Link between chronic inflammation and human papillomavirus-induced carcinogenesis (Review)

**DOI:** 10.3892/ol.2015.2884

**Published:** 2015-01-16

**Authors:** JOSÉ VERÍSSIMO FERNANDES, THALES ALLYRIO ARAÚJO DE MEDEIROS FERNANDES, JENNER CHRYSTIAN VERÍSSIMO DE AZEVEDO, RICARDO NEY OLIVEIRA COBUCCI, MARIA GORETTI FREIRE DE CARVALHO, VANIA SOUSA ANDRADE, JOSÉLIO MARIA GALVÃO DE ARAÚJO

**Affiliations:** 1 Department of Microbiology and Parasitology, Federal University of Rio Grande do Norte, Natal 59072-970, Brazil; 2 Department of Biomedical Sciences, University of Rio Grande do Norte State, Mossoró 59607-360, Brazil; 3 Department of Pediatrics, University Hospital Onofre Lopes, Natal 59072-970, Brazil; 4 Januário Cicco Maternity School, Federal University of Rio Grande do Norte, Natal 59072-970, Brazil; 5 Department of Pathology, Potiguar University, Natal 59020-500, Brazil

**Keywords:** inflammation, carcinogenesis, human papillomavirus, cervical cancer

## Abstract

Inflammation is a defense strategy against invading agents and harmful molecules that is activated immediately following a stimulus, and involves the release of cytokines and chemokines, which activate the innate immune response. These mediators act together to increase blood flow and vascular permeability, facilitating recruitment of effector cells to the site of injury. Following resolution of the injury and removal of the stimulus, inflammation is disabled, but if the stimulus persists, inflammation becomes chronic and is strongly associated with cancer. This is likely to be due to the fact that the inflammation leads to a wound that does not heal, requiring a constant renewal of cells, which increases the risk of neoplastic transformation. Debris from phagocytosis, including the reactive species of oxygen and nitrogen that cause damage to DNA already damaged by the leukotrienes and prostaglandins, has an impact on inflammation and various carcinogenic routes. There is an association between chronic inflammation, persistent infection and cancer, where oncogenic action is mediated by autocrine and paracrine signals, causing changes in somatic cells under the influence of the microbial genome or of epigenetic factors. Among the infectious agents associated with cancer, certain genotypes of human papillomavirus (HPV) stand out. HPV is responsible for virtually all cases of cervical cancer and a lower proportion of cancers of the vagina, vulva, anus, penis and a number of extragenital cancers. In the present review, recent advances in the mechanisms involved in the inflammatory response are presented with their participation in the process of carcinogenesis, emphasizing the role of chronic inflammation in the development of HPV-induced cervical cancer.

## 1. Introduction

Inflammation is a universal phenomenon that works during the occurrence of disturbances in homeostasis caused by infections, injury and exposure to irritants. The inflammatory reaction begins when molecular signaling pathways associated with pathogens or tissue damage are detected by the receptors of the innate immune system (1). A distinctive characteristic of the inflammatory response, in relation to other mechanisms of defense, is that during inflammation, damage to the host is inevitable. Despite the collateral damage that can result in pathologies, inflammation seeks the balance between tissue damage and self-maintenance (1,2). The challenge is to balance the immune response, so that the offending agent is removed with minimal damage, which is known as disease ecology (3).

From the physiological point of view, the function of inflammation is to quickly destroy or isolate the source of the disturbance, repair the damage incurred and restore homeostasis, being, therefore, a natural adaptation. One piece of evidence for this is the increased risk of serious infections in humans with genetic disorders in the primary components of inflammation. The same occurs in animals with deficient expression of genes encoding pro-inflammatory cytokines (4). By contrast, the deregulation of immunologically relevant genes leads to spontaneous inflammation, suggesting that, under normal conditions, the inflammatory response is suppressed to maintain health (5). The lack of control leads to exaggerated inflammation that can have devastating effects and cause severe pathologies (1).

Acute inflammation is quickly resolved following the removal of the stimulus and the repair of the tissue, but if the disturbance persists for a long time without repair, inflammation becomes chronic. This requires a constant cell renewal, which, in turn, leads to the acceleration of cell division and an increased risk of mutation and neoplastic transformation. Persistent infection induces chronic inflammation, where products generated by leukocytes and other cells, including leukotrienes and reactive species of oxygen and nitrogen, can cause DNA damage. Inflammatory cells also release prostaglandins produced by the action of enzyme cyclooxygenase-2 (COX2), which intensifies the inflammation and has an impact on various carcinogenic routes (6).

Clinical and epidemiological studies have revealed that certain pathogens that cause persistent infections are strongly associated with cancer (6,7). The oncogenic action appears to be mediated by autocrine and paracrine signals that are mediated by chronic inflammation, or by somatic alterations in the cells induced by the microbial genome (8).

The persistent infection with high-risk human papillomavirus (HR-HPV) is clearly defined as necessary for invasive cervical cancer (ICC) development. Once integrated with the host cell genome, HPV can cause genetic rearrangements, deletions, chromosomal inversions and translocations, activation of proto-oncogenes and loss of heterozygosity, which generates genomic instability and increases the risk of neoplastic transformation (9). However, the infection itself is not sufficient for transformation of the infected cell, unless it is accompanied by additional epigenetic events, including inflammation, facilitated by exposure to environmental factors or due to altered immunological mechanisms (10–14). In the present review, the recent advances about of the molecular and immunological mechanisms involved in the inflammatory reaction are reported, as well as the role of chronic inflammation in the development of HPV-associated cancer.

The literature search was conducted using the electronic databases PubMed (National Institutes of Health, Bethesda, MD, USA; www.ncbi.nlm.nih.gov/pubmed), Scopus (Elsevier, Amsterdam, Netherlands; www.scopus.com/scopus/home.url) and Web of Knowledge (Thomson Reuters, New York, NY, USA; wok.mimas.ac.uk), using the following keywords: Carcinogenesis, inflammation, inflammation and cancer, inflammation and HPV infection, and inflammation and cervical cancer. The databases retrieved thousands of articles and those that were thought to be most relevant were selected, which included recent studies published in journals of impact, conducted by groups with recognized expertise in the areas of inflammation, cancer and infectious agents, in particular HPV.

## 2. Mediators of the inflammatory response

Inflammation is a non-specific defense mechanism activated in response to tissue damage of any nature, and involves the innate and adaptive immune system combating the aggressor agents (15). This process consists of a complex cascade of events involving the participation of soluble mediators, termed cytokines and chemokines, which act collectively in the activation and regulation of the inflammatory response. Table I presents the main cytokines involved in the inflammatory response, the cells that produce them and the main mechanisms through which each operates during the activation and regulation of the inflammatory reaction (16–38).

## 3. Molecular mechanisms of the inflammatory response

Inflammation is a biological reaction that aims to correct disturbances in homeostasis occurring in the tissues by recruiting of leukocytes and other blood products*,* such as plasma proteins, to the site of injury. This migration is facilitated by vasodilation and increased vascular permeability and blood flow. Once activated, proinflammatory cytokines, including tumor necrosis factor (TNF), interleukin (IL)-1β and IL-6 are produced, but once the stimulus is removed, inflammation becomes naturally regulated and returns to its normal state (39). The reversion of inflammation to the state of homeostasis precedes a highly-regulated process, known as the resolution of inflammation, which is controlled by various anti-inflammatory mediators, particularly IL-10, transforming growth factor (TGF)-β and glucocorticoids, involving the recruitment of monocytes for the cleaning of cell debris (40).

The inflammatory response is a tightly regulated cascade of immunological, physiological and behavioral processes. The activation of inflammation begins when pattern recognition receptors (PRRs), which are expressed in the cells of the innate immune system, detect the presence the conserved structures of harmful agents, termed pathogen-associated molecular patterns (PAMPs), or endogenous molecules derived from lesions, termed damage-associated molecular patterns (DAMPs) (41,42). The PRRs are transmembrane receptors of various types, including Toll-like receptors (TLRs), C-type lectin receptors, RIG-like receptors, nucleotide-binding oligomerization domain-like receptors and rich-leucine receptors, which have a selective ability to detect PAMPs, DAMPs or the two together (43,44).

Once they have recognized PAMPs or DAMPs, the PRRs bind to target molecules and trigger common signaling pathways, culminating in the activation of nuclear factor κ of B cells (NF-κB), which is transported to the nucleus of the cell. In the nucleus, NF-κB activates the transcription of various genes, whose products instantly trigger the inflammatory response through the production of cytokines, chemokines and other mediators involved in the inflammatory process (42,45). In the case of viral infections, the signaling pathway involves the use of interferon type 1 (IFN-1) and the activation of cytotoxic T lymphocytes (CTLs). IFN-1 induces the phosphorylation and transport to the nucleus of a complex termed interferon-stimulated gene factor 3, which is composed of signal transducers and activators of transcription (STAT)-1 and -2, and IFN regulatory factor 3 (IRF-3). In turn, this activates the expression of antiviral genes, including protein kinase R (PKR) and 2′-5′-oligoadenylate synthetase (OAS). Thus, the proliferation of virus-infected cells is inhibited by PKR, while OAS suppresses viral replication by the cleavage of nucleotides (46,47).

Soluble factors, including IL-6 and macrophage colony-stimulating factor-1 (CSF-1)*,* derived from neoplastic cells, induce differentiation in myeloid cells to produce a similar phenotype as a macrophage. These cells are known as tumor-associated macrophages (TAMs) and they are the major components of inflammatory infiltrates in tumor tissue, and are recruited by monocyte chemotactic protein and other mediators (48). M2-polarized TAMs are key regulators of the link between inflammation and cancer. However, these cells have a contradictory role in neoplasia. TAMs can kill tumor cells following activation by IL-2, IFN-γ and IL-12, but they also produce IL-10, which inhibits the CTL-mediated antitumor response and therefore promotes tumor progression (5,48,49).

Chronic inflammation is due to the action of various inflammatory mediators, including TNF-α, IL-6 and IL-17, which suppress antitumor immunity and favor tumor progression. By contrast, cytokines, including TNF-related apoptosis-inducing ligand (TRAIL), IL-10 and IL-12, exert antitumor action. TRAIL exerts antitumor effects by inducing tumor cell apoptosis, IL-10 exerts antitumor effects through its anti-inflammatory action and IL-12 exerts antitumor effects by activating CTLs and natural killer (NK) cells and through the expression of cytotoxic mediators, which can lead to tumor suppression (50). Thus, cytokines secreted by tumor and inflammatory/immune cells can either promote tumor development and tumor cell survival, or they can exert antitumor effects, depending on the context in which they operate. Upon infection by pathogens, activated myeloid cells produce proinflammatory and anti-inflammatory cytokines. In addition to its direct effects on tumor growth, survival of tumor cells and invasive properties, cytokines can govern the functions of T helper (Th)1 cells, NK cells, regulatory T cells (Tregs) and Th17 cells, all of which can infiltrate into tumors. Treg-mediated suppression of antitumor CTL responses and induction of inflammatory Th17 cell-mediated responses contribute to tumor progression. Paradoxically, IL-10 can mediate the antitumor effects of Tregs. IL-23, TGF-β, IL-6 and TNF-α promote the development of Th17 cells, which play a central role in the coordination of chronic inflammatory responses and, thus, can contribute to the growth of tumors. IL-23 induces the release of IFN-γ and IL-12 from activated T cells, TNF-α and IL-12 from antigen presenting cells (APCs), and IL-17 from Th17 cells (51). However, it has been revealed that IL-23 inhibits IL-12 activity, particularly the activation of IFN-γ (51).

In contrast to innate immunity, whose role is well defined, little is known about the involvement of adaptive immunity in the inflammatory response, although it has been documented in several studies (52–54). The Th cells represent a specific population of lymphocytes of the adaptive immune response and are largely responsible for activating and regulating this response. When stimulated by APCs, Th cells that were never exposed to the antigen, termed naive T or Th0 cells, can differentiate into effector cells with a Th1, Th2, Th17 or Treg phenotype. The Th1 phenotype exhibits proinflammatory action, Th2 exhibits anti-inflammatory action, Treg exhibits regulatory action and Th17 is involved with intense inflammatory responses and autoimmunity (55). Th1 cells activate cellular immunity and induce a proinflammatory response against intracellular pathogens through the release of IFN-γ, which has potent antiviral properties and promotes differentiation of Th0 in Th1 cells. Th1 cells also secrete IL-2, IL-12 and TNF-α, all of which are important in mediating the response of delayed-type hypersensitivity and activation of macrophages (56).

By contrast, Th2 cells are important for humoral immunity by activating the proliferation of B lymphocytes, regulating allergic responses and protecting against infection by macroparasites such as helminthes (1). Th2 cells produce anti-inflammatory cytokines, including IL-4, -5, -10 and -13, which further stimulates the differentiation of Th0 to Th2, promotes alternative activation of macrophages and induces the differentiation of B lymphocytes, which produce and secrete antibodies, in plasma cells (56). Th2 cells also induce immunoglobulin class switching for immunoglobulin (Ig)E and eosinophil maturation, and negatively regulate the production of Th1 cytokines. Indeed, Th1 and Th2 responses are antagonistic and represent a balance between proinflammatory and anti-inflammatory mechanisms, where one can be selected and influence the outcome of infection (57). The ideal condition would be a balance between the Th1/Th2 phenotypes to promote elimination of the pathogen with minimal inflammation and damage to host tissues. Animal studies have revealed a limited capacity of the host to mount Th1 and Th2 responses that are each effective (1,56,57).

The notion of a simplified bimodal immune response to infection that activates the Th1 or Th2 phenotype was modified by the discovery of the existence of a cross-regulation by a T cell type, termed regulatory T cells (Treg). Treg cells are important to suppress the activation, proliferation and effector functions of various immune cells, including T lymphocytes, NK cells, B lymphocytes and APCs (57). Although the role of Treg in regulating the homeostasis of the immune system has not yet been elucidated, there is evidence that these cells appear to dampen Th1 and Th2 immune responses by the secretion of IL-10 and TGF-β. These cytokines have immunosuppressive action and inhibit the proliferation of Th1 and Th2 cells, presumably to minimize tissue damage. Treg cells also appear to play a role in mediating chronic infection, avoiding the inflammation of immune privileged organs (58). This indicates that the inhibition of Treg function results in an immunopathological response with damage to tissues. However, an increase of these cells can increase the chance of survival of the pathogen and, in certain cases, leads to long-term chronic infection (59). Thus, establishing a good balance between effector and regulatory functions so that the host can efficiently deal with the pathogen is the big challenge. Th17 cells represent a population of effector Th cells, which are largely involved in the elimination of extracellular microparasites, a process that requires an intense inflammatory response and is not properly dealt with by Th1 or Th2 cells (60).

## 4. Persistent infection, chronic inflammation and carcinogenesis

In its broader definition, inflammation is a process that involves events of tissue remodeling caused by changes in the functions of the cells of the vascular and immune systems. Such changes are orchestrated by specific molecular pathways that involve a number of cytokines, chemokines, growth factors and lipid mediators (61). Compelling evidence indicates that the majority of cancers arise from sites of chronic irritation, infection and long-term inflammation, solidifying the concept that chronic inflammation decompensated is a critical factor for the progression of tumors (6).

The connection between chronic inflammation and cancer does not only operate in one direction. Studies have revealed that DNA damage may also result in inflammation, since inflammation and DNA damage induce cell death by necrosis, which leads to more inflammation (62,63). Oncoproteins that are associated with cancer, including Ras, Myc and RET, can also cause inflammation via the activation of signaling mechanisms involved in the production of proinflammatory cytokines and chemokines (64). The main molecular link between inflammation, promotion and tumor progression is provided by association with IK/NF-κB, involving a signaling pathway that is activated by numerous proinflammatory cytokines (65,66). NF-κB is a transcription factor that regulates the expression of several genes that may suppress cell death through apoptosis and the stimulation of cell cycle progression in tumor cells. In addition, NF-κB promotes epithelial mesenchymal transition, which plays an important role in invasiveness as it provides the emerging tumor with an inflammatory microenvironment that supports tumor progression and invasion of the surrounding tissue, causing angiogenesis and metastasis (67–69).

HPV is a sexually transmitted agent deemed a cause of cervical intraepithelial neoplasia (CIN) and invasive cervical cancer (ICC) worldwide. Currently it is widely accepted that specific genotypes of HPV are potentially oncogenic and are associated with virtually all cases of cervical cancer and, to a lesser extent, with cancers of the vagina, vulva, anus, penis, skin and oropharynx (10,70–72). In general, HPV infection begins with viral replication in the cells of the germinal layer of the cervical epithelium. The viral replication continues in parallel with the differentiation of cells of the squamous epithelium, but assembly of virions only occurs in cells of the superficial layer of the epithelium that have reached the maximum degree of differentiation (73). In the majority of cases, the genital HPV infection is transient and heals spontaneously within 30 months. However, in certain individuals, the viral clearance does not occur during this period, the infection becomes persistent and can result in lesions that may eventually progress to cancer (74,75). Worldwide, cervical cancer is the second most common type of cancer in women, excluding non-melanoma skin cancer (76). The analysis of the pathogenesis of genital HPV infection and of the lesions associated with it, based on morphology, molecular biology, and epidemiology, revealed the existence of the infection without any lesion, with the presence of precursor lesions of low or of high-grade and invasive cancer (73). In the transition of HPV infection without lesions to cervical cancer, there is a gradual worsening of cytological changes characterized by increased nuclear atypia and a disorganized differentiation of the cervical epithelium, without invasion of the basement membrane, in the case of premalignant lesions or with invasion of the basement membrane in cases of cancer (75).

Persistent infection with HPV genotypes with high oncogenic potential increases the probability of ICC following an extended period of latency. The integrity of the cell-mediated immune response against HPV is an important factor for healing and prevention of the reactivation of the latent infection (75,77). Epidemiological studies in HPV-infected females have provided important clues about a spectrum of cofactors that can increase the carcinogenic potential of HPV. Presumably, cervical infections with other pathogens, exposure to physical and chemical agents, hormonal factors, nutritional status and genetic inheritance can serve as cofactors influencing the acquisition of HPV infection, as well as influencing the development of viral persistence (11,77). Certain studies suggest that other sexually-transmitted viral or bacterial agents may serve as cofactors for the development of HPV-associated cancer. These agents include the herpes simplex virus type 2 (HSV-2) (11,78) and *Chlamydia trachomatis* (79,80). Notably, the infections caused by these two pathogens are often associated with an intense chronic inflammatory response and ulcerations in the cervical epithelium (81,82).

## 5. microRNAs (miRNAs) inflammation and cancer

miRNAs are small non-coding RNAs that regulate the translation and degradation of target messenger RNAs (83). miRNAs have been implicated in the regulation of almost all aspects of cellular functions, including the innate and adaptive immune responses (84). miRNAs participate in numerous regulatory networks of genes whose dysfunctions are associated with diseases such as cancer (85). Several miRNAs have been implicated in inflammation and cancer through the activation of oncogenic and immune signaling pathways that converge in the expression of genes involved in activation of inflammation and carcinogenesis. Among these genes, the most important are miR-21, -125b, -155, -182, -196b and -210, which are known to be a source of immune and inflammatory stimuli (86).

The expression of miR-21, miR-155 and miR125b is controlled by an undetermined amount of immune signals, with TLR, TNF-α and cytokines being the most prominent signals that involve these miRNAs in inflammatory events (86). miR-155 expression is induced synergistically in NK cells following co-stimulation with IL-12 and IL-18 or only with IL-12. High levels of miR-155 expression enhance the induction and secretion of IFN-γ through the activation IL-12 and IL-18, whereas reduced miR-155 expression has the opposite effect (87). miR-21 is upregulated, *in vitro* and *in vivo*, by the oncogenes Ras or Src, the most frequently activated in human cancers (88). The mechanisms used by miRNAs to promote the initiation and progression of tumors include mechanisms that affect the modulation of by TLR, cytokines and their signaling pathways. Since cytokine signaling is essential for the differentiation of different cell subsets of the immune response, the miRNAs act as a limiting signal for differentiation these cells by modulating the expression of cytokines and their signaling components (86).

miRNAs also play an important role in the development of cancers associated with infectious agents. Infection with various pathogenic agents induces changes in the expression of miRNAs functionally related to the mounting of the innate immune response, which regulates the survival and proliferation of immunocompetent cells and controls the infections. Regardless of whether the infectious agent is bacterial, viral or parasitic, the expression of miR-21, miR-125, miR-155 and miRNAs is the most commonly affected during infection and, therefore, they have a potential role in infection-induced carcinogenesis. It has been demonstrated that upregulation in the expression of miR-21 and miR-182 is associated with HR-HPV-associated carcinogenesis (14,86).

## 6. B lymphocytes mediate chronic inflammation

Using an HPV16-expressing transgenic mouse model, de Visser *et al* (89) analyzed the interaction between innate and adaptive immunity in the progression of the tumor induced by the virus. The authors created a lineage of HPV16-expressing mice, with an inability to produce B and T cells, by crossing a line transgenic for HPV16 with a lineage deficient for B and T cells. The genetic elimination of the adaptive immune response blocked the recruitment of the cells of the innate immune response, as well as blocking the remodeling of tissue, angiogenesis and carcinogenesis. When B cells obtained from transgenic HPV16-expressing animals were transferred to the T and B cell-deficient HPV16-expressing animals, the inflammatory response and cancer progression were restored, demonstrating that B cells are responsible for this phenomenon. When B cells are not infiltrates in precancerous tissues, it is hypothesized that they should remotely mobilize the cells of the innate immune response. This was confirmed by the finding that the serum-free cells obtained from transgenic animals also restored the progression of cancer in animals deficient in T and B cells (89).

The absence of B cells at the lesion site does not exclude the possibility of the involvement of antibodies directed against molecules expressed on the surface of tumor cells. Therefore, this phenomenon could be attributed to soluble mediators that promote interactions with APCs derived from the skin with the drainage of these cells to the lymph nodes and/or spleen where they would be able to trigger chronic inflammation. With regard to the type of soluble mediator produced by activated B cells at a distance that could be involved in the activation and maintenance of chronic inflammation in mice transgenic for HPV16, it has been reported that B cells produce chemokines including chemokine (C-X-C motif) ligand (CXCL) 1 and keratinocyte chemoattractant, in addition to cytokines IL-4 and IL-10 (90). These mediators have been implicated in the suppression of innate immunity during the assault by pathogens and/or in acute inflammation (91). Thus, inflammation could be involved in mediating the recruitment of leukocytes to neoplastic skin. The mechanisms of participation of these mediators entails the formation of an antigen-antibody complex, the activation of the complement and release of the anaphylatoxins C3a and C5a, which are potent proinflammatory factors. Alternatively, antibodies can activate the innate immune response through direct involvement by binding to multimeric Fc receptors present on the cell surface. Each case would lead to an inflammatory reaction (89). The genetic elimination of T and B cells does not increase the incidence of progression of premalignant lesions to HPV-associated carcinoma, on the contrary, it reduces the risk of this occurring, which suggests that adaptive immunity somehow participates in the process of chronic inflammation and carcinogenesis (89). This contradicts what has been observed in clinical and epidemiological studies, where it has been revealed that immunosuppressed humans are at higher risk for certain types of malignancies, including non-Hodgkin’s lymphoma induced by Epstein-Barr virus and Kaposi’s sarcoma associated with Kaposi’s sarcoma-associated herpes virus (8,89). However, the relatively high incidence of these malignancies in immunocompromised patients may be at least partly explained by the greater susceptibility to infection and/or reactivation of latent infections caused by these viruses (8).

## 7. Chronic inflammation and HPV-induced cancer

Currently there is no more doubt that the persistent infection of the cervical mucosa with high-risk HPV (HR-HPV) is a necessary cause for the development of squamous intraepithelial neoplasia (SIL), low-grade SIL (LSIL) high-grade SIL (HSIL) and invasive cervical cancer (ICC). However, numerous women are infected with HR-HPV, but the majority of them never develop disease, suggesting that other factors must contribute to the development of the lesions and the transition from premalignant to malignant lesions. Prior to the discovery of the HPV, infection with *C. trachomatis* and HSV-2 were considered to be major risk factors for the development of ICC. However, it is now known that this association had probably been mistaken for a long time, due to a lack of methods able to detect the presence of HPV in tumor cells (91).

In past decades, studies have reported an association between chronic inflammation and the occurrence of HSIL and ICC (92–94). Furthermore, it was demonstrated that in infected females with HR-HPV, the presence of HSIL was highly associated with inflammation. This reinforces the concept that inflammation may be an important cofactor, contributing with HPV in the development of HSIL (95,96). Furthermore, was also demonstrated that seminal fluid contains a variety of active molecules, including cytokines, angiogenic factors, proteases, protein kinases, carrier proteins, structural proteins and molecules of the immune response, which are associated with inflammation (97). Considering that the development of HSIL and ICC requires an inflammatory environment, it has been proposed that these mediators present in seminal fluid could act to cause inflammation in the cervical mucosa, increasing the risk of lesions. Thus, it is possible that the deposition of seminal fluid in the mucosa of the female genital tract triggers a wave of cytokine release, with the recruitment and activation of leukocytes resulting in inflammation (98). This is consistent with previously reported data, which revealed that the use of condoms during sexual intercourse, despite not offering significant protection against genital infection by HPV, significantly reduces the occurrence of lesions in cervical mucosa caused by this virus (99). This mucosal protection offered by condoms against HPV infection, is likely due to them preventing contact between the semen and the epithelium of the cervical mucosa.

Notably, Schwebke and Zajackowski (93) hypothesized that inflammation of the uterine cervix, but not the diagnosis of a specific sexually transmitted disease, is associated with an increased risk of SIL and ICC. However, it is likely that the increased risk of cancer associated with chronic inflammation is due, at least in part, to the presence of infection with other sexually transmitted agents, including HSV-2, *C. trachomatis* and others, that cause an intense local inflammatory response (11,89). Various studies have demonstrated that the development of cancer induced by HR-HPV is preceded by chronic long-term inflammation, which in numerous cases, is developed with the participation of other sexually transmissible infectious agents, including HSV-2 and *C. trachomatis* (81,82,100,101).

Histological analysis of biopsies of cervical mucosa from HR-HPV-infected females revealed a higher degree of inflammation, with an increased infiltration of lymphocytes and neutrophils into the epithelium, compared with those who were uninfected. The association between persistent infection with a specific HPV type and progression of LSIL to HSIL was less evident in women with moderate inflammation in the stroma or in the epithelium. In individuals infected with HR-HPV, the inflammation tended to involve only the surface layer of the epithelium, instead of the basal layer, or involvement extended to all the cervical epithelium (96). This suggests that inflammation varies with HPV type, persistent or not, and possesses a risk of progression.

Assessing the number of macrophages present in the cervical epithelium of healthy females and those with LSIL, HSIL and ICC, Hammes *et al* (96) identified a positive linear association between the amount of macrophages and the progression of lesions, finding that an increased migration of macrophages to the epithelium was proportional to the worsening of the injury. A direct association between the amount of macrophages present in the epithelium and the progression of premalignant lesions to cancer was also identified. The intensity of inflammation was also closely associated with the degree of the lesion. The macrophage migration from the epithelium to the stroma was influenced by not only inflammation, but also by the presence of dysplastic cells. These data reveal that cluster of differentiation (CD)68^+^ macrophages are associated with cervical carcinogenesis, promoting the progression of intraepithelial lesions to invasive forms.

The role of the E6 and E7 HPV16 oncogenes in the regulation of IL-1β expression was analyzed by Niebler *et al* (102) in HPV16-immortalized human keratinocytes and in cells obtained from cervical cancer. These researchers found that in keratinocytes expressing only E7, the secretion of IL-1β was highly inducible by the activation of inflammation; however, in those expressing only E6, no production of IL-1β was detected and the IL-1β precursor and p53 were degraded in a proteasome-dependent manner, mediated by ubiquitin ligase E6-AP. By contrast, in keratinocytes expressing E6 and E7 or only E7, the levels of the IL-1β precursor were restored by interference RNAs, which block the expression of E6-AP, with simultaneous recovery of functional p53. The results from this study indicated the presence of a novel mechanism for post-translational regulation of the IL-1β precursor that inhibits IL-1β secretion in HPV-infected keratinocytes. This reveals an effective and innovative mechanism of HR-HPV to prevent the function of IL-1β, which reduces the innate immune response against the infected cells, facilitating persistence of the virus, which is an important step in the initiation of the cell transformation and tumorigenesis (102).

The systemic levels of proinflammatory cytokines in female patients with persistent HPV infection were evaluated by Kemp *et al* (103), who found significantly elevated levels of IL-6, IL-8, TNF-α, macrophage inflammatory protein (MIP)-1α, granulocyte-macrophage colony-stimulating factor, IL-1α and IL-1β in the plasma of the patients with the infection compared with uninfected individuals. It was also noted that high systemic levels of these cytokines were accompanied by a reduction in lymphoproliferative response. This is a novel finding and is somewhat unexpected as there is little evidence of an HPV-induced systemic inflammatory reaction. The presence of proinflammatory cytokines in the peripheral blood was associated with HPV persistence and weak lymphoproliferative response (103).

Analyzing the profile of immune response in a case-control study, Peghini *et al* (13), found that 75% of individuals with normal cytology exhibited a profile of Th1 cytokines, compared with only 10% of women with LSIL, HSIL or ICC. The samples of ICC exhibited more commonly a Treg profile and behaved in the opposite way to Th1. Changes in the profile of cytokines present in the microenvironment were also revealed during the progression of lesions, demonstrating a significant reduction of IL-2, IL-12 and TNFα, and an increase in TGFβ with aggravation of the lesions. The pattern of cytokines found during tumor progression makes it clear that there is involvement of Treg cells in the induction of ICC. Tumor progression was dependent on the suppression of cellular immunity since normal individuals or individuals with less severe lesions were observed to possess a profile of Th1 cytokines, with a change to an immunosuppressive Treg profile in those with neoplastic progression (13). This suggests that a microenvironment with chronic inflammation and an imbalance in the Th1/Treg ratio and the levels of cytokines produced appears to be a critical mechanism for tumor-cell evasion of the immune surveillance system, facilitating tumor progression.

The exact mechanism of the clearance of HPV infection remains unclear. HPV persistence is considered to require a tolerant local immune environment, favoring evasion of the virus or the suppression of the innate and adaptive immune responses (103). The absence of viremia and cytolysis in cervical HPV infection contributes to the difficulty in defining the immunological mechanisms regulating the clearance of the virus (76,104). It is thought that the innate immune response has a critical role early in the process of healing of HPV infection (105).

Analysis of the signaling of PRRs in uninfected and newly infected keratinocytes, and those persistently infected with HR-HPV, revealed that active infection impairs emission of signals from PRRs to the cell nucleus, affecting the production of type 1 IFNs, proinflammatory cytokines and chemokines. This mechanism of suppression is dependent on the expression of the intracellular protein ubiquitin carboxyl-terminal L1 (UCHL1), which is induced by HR-HPV in keratinocytes. UCHL1 mediates the degradation of NF-κB and promotes phosphorylation of p65, resulting in the chronic suppression of NF-κB signaling, an important modulator of cellular immunity. This indicates that HR-HPV uses UCHL1 to escape the innate immunity of the host, by suppressing the production of interferons, cytokines and chemokines induced by PRRs, which are necessary for the attraction and activation of cells that are also involved in the adaptive immune response (106).

Notably, following the integration of HR-HPV into the genome of the host cell, NF-κB appears to go on to have an important role in the development of ICC, since this transcription factor was detected in nucleus and cytoplasm of 96.4% of HPV-positive tumor samples and in only 52.9% of HPV-negative tumor samples. The overexpression of NF-κB in ICC suggests that NF-κB exerts modulating effects on chronic inflammation and increases the risk of cancer. The mechanism of action of NF-κB in the process of HPV-induced cervical carcinogenesis is probably due to the action of the viral oncoproteins E6 and E7 that, besides increasing the transcriptional activity of NF-κB, eliminate the functions of cellular proteins p53 and pRB, leading to an uncontrolled cell cycle with induction of immortalization and neoplastic transformation of the cell (107). It has been demonstrated that E7 induces expression of IL-1β which, in turn, activates the expression of NF-κB, while E6 is associated with the nuclear localization of this transcription factor. Thus E6 and E7 are associated with the increased activity of NF-κB that is a key modulator in the transition from chronic inflammation to cancer (108,109).

In a recent multiethnic cohort study, Scott *et al* (110) monitored females between 2005 and 2010 to evaluate the expression levels of candidate antiviral cytokines in mucosa, including IFN-α2, IFN-γ, IL-12 and IL-10, as well as the proinflammatory cytokines (IL-1α, -1β, -6 and -8, CXCL8, MIP-1α, CCl3 and TNF-α) that are induced following the establishment of HPV infection. The majority of the cohort exhibited increased expression of certain cytokines and decreased expression of others following infection. An association was observed between high levels of IL-10, IL-12, MIP-1α and TNF-α in the cervical mucosa and a reduced likelihood of clearance of any HPV type. For HR-HPV, the trend of no viral clearance was significantly higher in the presence of high levels of IL-12 and TNF-α. Similar patterns were observed in infection with low-risk HPV, although the trend of not healing was more modest, revealing a significant difference only for IL-10. In the group of women who had rapid healing of HPV infection, low levels of cytokines were associated with a more rapid clearance of the virus, compared with the group of women with established long-term infection. The clearance of HR-HPV was more frequent in the presence of low levels of MIP-1α and IL-8 expression in the cervical mucosa in the group of women that demonstrated rapid healing of infection, but not in the group with established long-term infection. Increased local levels of several important proinflammatory cytokines, including TNFα, MIP-1α, IL-12 and IL-10, were associated with a decreased likelihood of elimination of the virus in women with established long-term infection (110). Scott *et al* made two considerations regarding the results. The first is that in individuals with transient exposure to the virus, in the absence of productive infection, the induction of cytokines may not occur and the virus can be eliminated by a non-immune mechanism. This would explain the absence of high levels of cytokines among women with rapid clearance of the virus. The second consideration is that, in a successful immune response, the immune mechanisms of homeostatic control may precede the elimination of the virus, bringing the previously high cytokine levels back to baseline levels in anticipation of the elimination of the virus.

There is evidence to support a significant role of TNFα in the immune response against HPV infected cells, as well as in the natural history of disease associated with this virus, since expression of this cytokine by infiltrating mononuclear cells is correlated with spontaneous regression of the lesions induced by HPV. Furthermore, it has been demonstrated that the HPV E6 and E7 proteins suppress the protective effect of TNFα, as E6 induces resistance to TNFα-mediated apoptosis (110) and E7 inhibits the antiproliferative effect of this cytokine (109). This suggests that acquisition of resistance to TNFα may be an important step in HPV-induced carcinogenesis. However, there is also evidence that, under certain conditions, TNFα can act as a tumor promoter (111). This is an eloquent demonstration of the complexity of the interactions established between the broad spectrum of cytokines produced during the inflammatory response, in which certain cytokines have effects and functions that are occasionally contradictory, depending on the context in which they operate.

Persistent infection with HR-HPV results in the integration of viral DNA into the host cell genome and overexpression of oncogenes E6 and E7, which are considered key factors for the development of ICC. These viral genes encode the E6 and E7 oncoproteins, which are responsible for the transforming activity of infected cells. The main targets of these viral proteins are the cellular tumor suppressor proteins p53 and pRB, which are responsible for cell cycle control (112–114). Though oncogenes E6 and E7 are major HPV genes involved in neoplastic transformation, evidence points to an important role of the E5 viral oncogene in tumorigenesis and in modulation of immune cells (115). E5 is also involved in the regulation of late viral functions, together with viral gene E4. The E1 and E2 viral genes encode viral replication factors and appear to play a role in HPV persistence, enabling copies of the virus to be maintained in an episomal form in the nucleus and be transferred to daughter cells during mitosis (73,116). Within the epithelial cells, the HPV E6 protein binds to the cellular protein p53 and promotes its degradation by an ubiquitin-dependent pathway, while the HPV E7 protein binds to the pRB family members, consisting of p105, p107 and p130, and promotes their degradation. The elimination of the functions of p53 and pRB results in uncontrolled cell cycle progression and loss of DNA repair mechanisms, with the consequent accumulation of mutations creating genomic instability (72,73). Furthermore, the expression of HPV E6 and E7 genes in differentiating keratinocytes directly alters the expression of genes that influence host resistance to infection and immune functions. A primary function of E6 and E7 is to maintain the keratinocytes in the cell cycle, but they also prevent antiviral and antitumor effects, and exert immunoregulatory action of the IFN-mediated innate immune system. E7 protein blocks IFN-α activity by inhibiting the expression of the inducible genes that lead to IFN-α production. E7 also inhibits the production of IFN-β by inhibiting the activation of the IFN-β promoter (113). The changes in cellular functions triggered by the action of viral oncoproteins can also result in increased production of nitric oxide, DNA damage, and activation of cyclooxygenase-2/prostaglandin/prostaglandin receptors (COX2/PGE/PGERs), an inflammatory pathway that leads to increased inflammation and tumorigenesis. Inflammatory and tumor cells can then release cytokines, chemokines and prostaglandins which act in an autocrine/paracrine manner to regulate the function of endothelial, stromal, neoplastic, epithelial and infiltrating immune cells to cause increased tumor angiogenesis, increased tumor growth, decreased apoptosis and decreased local immune-surveillance. These conditions favor tumorigenesis and permanence of the virus in the tumor microenvironment. Inhibition of the COX-PGE cascade with nonsteroidal anti-inflammatory drugs such as aspirin could reduce inflammation and tumor progression (82,117).

The relationship between inflammation, angiogenesis and HPV-induced lesions was assessed by Mazibranda *et al* (12), in samples obtained from patients with normal cytology, CIN and ICC. Based on the density of pan-endothelial cells present on-site, identified by the CD31 marker, a progressive increase in microvessels was observed, with increasing severity of the lesion. Macrophage infiltration was associated with neovascularity in CIN and ICC, with a strong positive correlation between the number of infiltrating macrophages in the tumor and its vasculature. An increase in macrophages in microvessels was also observed in parallel with neoplastic progression, being even higher in ICC (12).

Immune evasion is an essential aspect of HPV persistence and is also essential for the development of ICC. As there is no sign of viremia, cytolysis or necrosis in the early-phase HPV-infected cervical epithelium, even when it is productive, there is not an adequate activation of the innate immune system or inflammation. This is due to the fact that the viral E6 and E7 oncoproteins inhibit the expression of PRRs, particularly TLR9, promoting dysregulation of the interferon signaling pathway, preventing activation of the innate immune response and allowing cells to remain infected with HPV, which uses the cellular machinery for viral replication followed by persistence (109,112). Once established, persistent HPV infection leads to changes in the release of proinflammatory cytokines, which in turn may alter the infiltration of immune cells, causing inflammation. Changes in immune response and elevated systemic levels of proinflammatory cytokines have been observed in older women, ~50 years old, with persistent HPV infection (111). It was demonstrated that in neoplastic epithelial cells of the cervical mucosa, the E5, E6 and E7 HPV16 oncogenes were able to induce the cyclooxygenase (COX)-prostaglandin inflammatory axis, increasing the immediate expression of the COX2 gene (82). These results suggest a direct link between the HPV oncogenes and activation of potent inflammatory cascades with well-known roles in cancer promotion. Thus, although HPV is not associated with inflammation at the initiation of infection, it is likely that, following its integration, persistence and transformation leads to the activation of inflammatory pathways, including the COX-prostaglandin pathway, in neoplastic epithelial cells, promoting immune cell infiltration, inflammation, and tumor progression (118). Biopsy studies of tumors associated with HPV reveal significantly higher expression levels of COX1 and COX2 in neoplastic epithelial cells and vascular endothelial cells of all grades and stages in ICC (110,109). These findings suggest an important role for the two isoforms of COX in the pathology of disease. Furthermore, it was revealed that the induction of COX1 expression in HeLa cells, a lineage positive for HPV18, caused a rapid and sustained elevation of COX2 and prostaglandin synthase terminal, resulting in the synthesis of PGE2 (109). Furthermore, PGE2 was produced by COX1 and COX2, indicating that they may contribute equally, or act synergistically, to promote ICC (118).

The mechanism for the selective production of prostaglandin is determined by terminal prostaglandin synthase, which depends on the PTGES synthase enzyme present in COX1- and COX2-expressing cells that converts prostaglandin H2 into PGE2 (98). It was demonstrated that this enzyme is represented significantly in ICC, and that products of the oncogenes of HPV and PGE2 can regulate the expression of the prostaglandin E receptor PTGER (119). Furthermore, it was demonstrated that the E5 protein of HPV16 regulates the expression of PTGER4 in the cells obtained from ICC, so that the production of PGE2 is cyclic adenosine monophosphate (cAMP) dependent (115). These results suggest that increased levels of PGE2 in ICC can regulate the function of neoplastic cells in an autocrine or paracrine manner, through the expression of high levels of the prostaglandin receptors PTGER2 and PTGER4.

There is evidence to indicate that the initial HPV infection is followed by integration of the viral DNA into the genome of the epithelial cells of the cervical mucosa and activation of viral oncogenes, leading to the induction of COX1 and 2 expression, PTGE synthase expression, PGE2 biosynthesis and PTGER expression. In turn, PGE2 can regulate the function of tumor cells through PTGER by the cAMP signaling pathway (118). Certain immunological parameters were evaluated in women infected with HR-HPV, without lesions, CIN1, CIN3, carcinoma *in situ* (CIS) and ICC. The results revealed that the cervical mucosa was infiltrated by B cells expressing the CD20 and CD138 markers as early as in CIN1 and this infiltration increased with the worsening of the lesion, being strongly correlated with the infiltration of lymphoid cells expressing the marker CD32B and lymphocytes expressing FoxP3. The lymphoid cells GATA3 and T-bet were found in greater amounts in samples from patients with ICC compared with those obtained from women with normal cytology. The expression levels of the cytokines thymic stromal lymphopoietin and indoleamine 2,3-dioxygenase-1, promoting Th2 response and serum levels of IL-10, in epithelial cells were higher in the samples from patients with CIN3/CIS and in ICC compared with cytologically-normal samples. An increased infiltration of CD138-, CD20- or CD32B-expressing lymphoid cells into the stroma were observed as early as in CIN1. This indicates that a Th2-type response is present from the first histological evidence of pre-neoplastic transformation caused by HR-HPV. Furthermore, the presence of stromal lymphocytes expressing FoxP3, a marker expressed primarily by Treg cells, was detected in CIN3/CIS and ICC (120).

In a case-control study, Chen *et al* (33) analyzed the cytokine profile and calculated the percentages of Treg and Th17 cells in the peripheral blood of females with normal cytology, CIN1 and ICC. They observed a significant increase in the levels of TGF-β, IL-6, -10, -17 and -23 expression, and lower expression levels of IFN-γ, in patients with CIN1 and ICC compared with those with normal cytology. These results suggest that the immune response in patients with ICC has been suppressed. It was also observed that the Th17/Treg ratio in the patients with CIN1 and in those with ICC was significantly higher than in the control group. These data suggest that in patients with ICC, the percentage of Th17 and the Th17/Treg ratio were significantly altered. Also, a positive correlation was found between the percentages of Th17 and expression levels of IL-6, -23 and -17, all of which were higher in patients with ICC. A positive correlation was also found between the percentages of Treg and expression levels of TGF-β and IL-6. By contrast, there was a negative correlation between the percentage of Treg cells and the expression levels of IFN-γ (33). These data indicate an imbalance in the TH17/Treg ratio in the peripheral blood of patients with ICC. In a previous study, Zhang *et al* (32) had already found the percentage of circulating Th17 and Treg cells to be significantly higher in patients with CIN and ICC compared with a healthy control group. This study also reported an important imbalance in the average Th17/Treg ratio in patients with CIN or ICC. Furthermore, IL-17 and IL-10 levels were significantly higher in patients with ICC compared with the control group. The levels of Th17 and Treg cells gradually increased during the progression of the disease, leading to an imbalance of the TH17/Treg ratio in patients with ICC, suggesting a potential role of the Th17/Treg imbalance in the progression of CIN to cancer.

## 8. Conclusion

Long-term chronic inflammation in addition to persistent HPV infection is one potential cause for the development of ICC, involving the participation of reactive species of oxygen and nitrogen, cytokines, chemokines, growth and survival cell factors, enzymes including COX and metalloproteinase, prostaglandins and specific types of microRNAs. The collective action of these mediators induces changes in the processes of proliferation, senescence and cell death, and also in mutation, methylation of DNA and angiogenesis, contributing to development of HPV-induced ICC. Persistent infection with HR-HPV leads to the integration of the viral DNA into the genome of the host cell, with the overexpression of the viral oncogenes E5, E6 and E7. The product of E5 promotes fusion between cells, causing aneuploidy, and favors the transforming activity of E6 and E7 and immune evasion of the virus. In addition, E5 reduces the expression of class I major histocompatibility complex and causes inflammation by increasing COX2 expression which, in turn, increases the secretion of prostaglandin and its receptors. The products of the E6 and E7 genes eliminates the function of p53 and intracellular pRB, resulting in a loss of control of the cell cycle and of DNA repair, favoring accumulation of mutations and increasing the risk of neoplastic transformation. E6 activates telomerase, preventing senescence and cell death, leading to immortalization of the cell. E7 induces the expression of IL1β, which, in turn activates the expression of NF-κB, a key modulator in the transition of chronic inflammation to cancer. E6 and E7 remain correlated with production of the matrix metalloproteinase enzyme, which acts in remodeling the vascular system, facilitating diapedesis and angiogenesis. The high levels of TGF-β and IL-6, -10, -17 and -23 and the low level of IFN-γ in patients with ICC compared with healthy females, suggesting that the immune response was suppressed in ICC patients. Also, the percentage of Th17 and the TH17/Treg ratio are significantly altered, which suggests that the microenvironment of chronic inflammation changes the cytokine profile and promotes an imbalance in Th1/Treg, that appears to be critical to tumor-cell evasion of the immune surveillance system. ICC favors cellular proliferation, inhibits apoptosis and promotes tissue remodeling and angiogenesis, increasing the risk of tumor progression and metastasis.

## Figures and Tables

**Table I:** Main cytokine mediators, the inflammatory response and their respective roles.

Cytokine	Producing cell	Main functions
IL-1β	Monocytes and macrophages	IL-1β exhibits proinflammatory action, participates in the recruitment and activation of the cells of the adaptive immune system and acts as a signal for the activation of nuclear factor kappa of B cells (NF-κB) (16–18).
TNF-α	Inflammatory and tumor cells	TNF-α stimulates the growth and survival of tumor cells, exhibits antiapoptotic action, facilitates tumor progression, promotes angiogenesis and decreases immune surveillance by suppressing the cytotoxic activity of T lymphocytes and activated macrophages (19).
TGF-β	Macrophages, T lymphocytes	TGF-β exhibits anti-inflammatory and immunosuppressive action, presenting a central role in the proliferation and regulatory function of Treg cells by inducing Fox3. TGF-β also inhibits T-cell proliferation, the activation of macrophages and blocks the effects of proinflammatory cytokines(20,21).
IL-6	Macrophages, T lymphocytes	IL-6 exhibits proinflammatory action and plays a central role in the adaptive immune response, in inflammation and in tumor progression due to its ability to promote cell proliferation, inhibit apoptosis and neutralize the cytostatic effects of TGF-β (22,23).
IL-10	Macrophages and Th2 cells	IL-10 inhibits the production of IFN-γ by Th1 cells and the production of cytokines by activated macrophages, inhibits the effects of IL-6 and IL-12. IL-10 also possesses antinflammatory and antiproliferative activity, therefore inhibiting the formation and progression of tumors (24,25).
IFN-α/β	Many cell types	IFN-α/β are key effectors of the innate immune response to viral infections, activating a signaling cascade that activates numerous genes whose products inhibit viral replication. IFN-α/β also regulate the production of IFN-γ and possess anti-inflammatory activity, through inhibiting the production of IL-1, -18 and -12 and increasing the production of IL-10 (26–28).
IFN-γ	APCs, NK, NKT and Th1 cells	IFN-γ Activates macrophages during the innate immune response, increases the activity of NK cells and the production of MHC class II by macrophages. Following the initiation of the adaptive immune response, IFN-γ upregulates the Th1-cell response and inhibits the response of Th2 cells (29,30).
IL-17	Th17	IL-17 plays a central role in the elimination of pathogens, but persistent production causes chronic inflammation, including the production of TNF-α, IL-1β and IL-6, which amplifies the inflammation. IL-17 is found in high levels in patients with cervical cancer (31–33).
IL-12	Activated APCs	IL-12 is involved in the differentiation of naive T cells into Th1, stimulates the production of IFN-γ and TNF-α by T and NK cells, reduces IL-4-mediated suppression of IFN-γ and increases the cytotoxic activity of NK and TCD8^+^ cells (34,35).
IL-23	Activated APCs	IL-23 acts in a paracrine manner by inducing macrophage production of TNF-α, DC production of IL-12 and IL-17 production, which promotes the end stage of inflammation. Despite the similarities between IL-12 and IL-23, they follow divergent immune pathways and exert distinct effects on the development of tumors (34,35).
IL-18	Macrophages and other cells	IL-18, together with IL-12, induces a Th1-type immune response. IL-18 induces NK and T cells to release IFN-γ, which activates macrophages and other cells. The combination of IL-18 and IL-12 inhibits IL-4 production and Th2 differentiations, and induces a severe inflammatory reaction (36–38).

IL, interleukin; TNF, tumor necrosis factor; TGF, transforming growth factor; IFN, interferon; MHC, major histocompatibility complex; APC, antigen-presenting cell; NK, natural killer, NKT, natural killer T; Th, T helper; TCD8^+^, T cell positive for cluster of differentiation 8 (cytotoxic T cell).
